# Augmenting pharmacotherapy with physical exercise: review of the principles

**DOI:** 10.1192/j.eurpsy.2024.1237

**Published:** 2024-08-27

**Authors:** A. R. Szczegielniak, R. Pudlo

**Affiliations:** Department of Psychoprophylaxis, Medical University of Silesia, Katowice, Poland

## Abstract

**Introduction:**

Observed structural and functional changes in the central nervous system as a result of physical exercise are beneficial from biological, psychological and social standpoint. The studies published so far confirm that physical exercise, understood as planned, ordered and repetitive activity, can improve severity of symptoms, general functioning, and quality of life in patients with mood disorders, schizophrenia/psychotic disorders, anxiety, PTSD or addictions. This seems to be particularly important in relation to the growing number of patients facing resistance to classical pharmacological treatment as well as its side effects (e.g. metabolic syndrome, cardiovascular complications).

**Objectives:**

Review of effective implementation of treatment programs based on physical exercise within mental health services.

**Methods:**

Scoping review was performed by identifying relevant studies available in the PubMed and Scopus databases that were 1) peer-reviewed 2) in English language 3) focused on physical exercises 4) published within the last 10 years. Selection of the studies from the initial group of search results was performed manually.

**Results:**

Majority of studies present programs covering relatively small, diverse groups of patients with mixed types of physical exercise modalities and intensity introduced, which makes generalization to basic principles very difficult. Needs assessment of patients from various diagnostic categories is vital in the process of implementation and evaluation. Barriers indicated by service users include lack of psychoeducation on perceived benefits, limitations within healthcare system (e.g., time limits, cost, access), side effects of medication, and psychosocial factors such as isolation. The assessment of factors engaging and motivating to maintain physical activity seems particularly important.

**Conclusions:**

Identification of patients that may especially benefit from the inclusion of physical exercise, and recognition of therapeutic programs’ elements that ensure the maintenance of the physical activity require further research.

**Disclosure of Interest:**

None Declared

